# Assessment of integration of emergency obstetric and newborn care in maternal and newborn care in healthcare facilities in Osun State, Nigeria

**DOI:** 10.1371/journal.pone.0249334

**Published:** 2021-04-15

**Authors:** Abiola Olubusola Komolafe, Adekemi Eunice Olowokere, Omolola Oladunni Irinoye

**Affiliations:** Department of Nursing Science, Obafemi Awolowo University, Ile-Ife, Osun State, Nigeria; University of Mississippi Medical Center, UNITED STATES

## Abstract

The integration of emergency obstetric and newborn care (EmONC) into maternal and newborn care is essential for its effectiveness to avert preventable maternal and newborn deaths in healthcare facilities. This study used a theory-oriented quantitative approach to document the reported extent of EmONC integration, and its relationship with EmONC training, guidelines availability and level of healthcare facility. A descriptive cross-sectional study was conducted among five hundred and five (505) healthcare providers and facility managers across the three levels of healthcare delivery. An adapted questionnaire from NoMad instrument was used to collect data on the integration of EmONC from the study participants. Ethical approval was obtained and informed consents taken from the participants. Both descriptive (frequency, percentage, mean and median) and inferential analyses (Kruskal Wallis and Mann Whitney tests) were done with statistical significance level of p<0.05 using STATA 14. The mean age of respondents was 38.68±8.27. The results showed that the EmONC integration median score at the three levels of healthcare delivery was high (77 (IQR = 83–71)). The EmONC integration median score were 76 (IQR = 84–70), 76 (IQR = 80–68) and 78 (IQR = 84–74) in the primary, secondary and tertiary healthcare facilities respectively. Integration of EmONC was highest (83 (IQR = 87–78)) among healthcare providers who had EmONC training and also had EmONC guidelines made available to them. There were significant differences in EmONC integration at the three levels of healthcare delivery (*p* = 0.046), among healthcare providers who had EmONC training and those with EmONC guidelines available in their maternity units (*p* = 0.001). EmONC integration was reportedly high and significantly associated with EmONC training and availability of guidelines. However, the congruence of reported and actual extent of integration of EmONC at the three levels of healthcare delivery still need validation as such would account for the implementation success and maternal-neonatal outcomes.

## Introduction

Maternal and newborn morbidity and mortality is a worldwide health challenge with burden disproportionately distributed and highest in developing countries. The 2015 estimates of global maternal and neonatal mortality documented 303,000 maternal deaths, 2.6 million stillbirths and 2.7 million newborn deaths, most of which happened in developing nations especially Sub-Saharan Africa [1]. Nigeria has been one of the major contributors to the world’s maternal and newborn deaths with 814 maternal deaths/100,000 births [2] and 38/1,000 live births formally documented [3]. From anecdotal observations, this may not be the full picture as some maternal and child complications and deaths from pregnancy and childbirth related care by untrained and inappropriately monitored birth attendants at the grassroots and rural communities go un-captured in formal national statistics.

Obstetric complications such as haemorrhage, sepsis, eclampsia, obstructed labour and fetal distress remain the leading cause of deaths in women of reproductive age and neonates in low and middle-income countries. These deaths are preventable with implementation of emergency obstetric and neonatal care (EmONC) to treat and manage the obstetric complications in healthcare facilities [4–8]. The Nigeria healthcare delivery system is at three levels of healthcare delivery namely the primary, secondary and the tertiary healthcare. The primary healthcare provides preventive care while secondary and tertiary healthcare provide curative care. The management of cases of obstetric complications are expected to happen across the three levels. The primary healthcare facilities at the grassroots are required to provide basic emergency obstetric and newborn care (BEmONC) while the secondary and tertiary healthcare facilities provide both BEmONC and comprehensive emergency obstetric and newborn care (CEmONC) [9]. Generally, healthcare facilities are classified as either basic or comprehensive EmONC healthcare facilities based on their ability to implement the EmONC signal functions [10]. The BEmONC healthcare facilities are expected to implement seven of the nine signal functions while the comprehensive emergency obstetric and newborn care CEmONC implement all the nine signal functions [11].

Nigeria as a nation has adopted EmONC as an evidence-based practice to be implemented in healthcare facilities to reduce maternal and neonatal morbidity and mortality, yet only 1.2% and 3.9% of public healthcare facilities fulfil the criteria for BEmONC and CEmONC respectively [12]. Ntambue et al. report that less than one-third of women with obstetric complications are admitted in facilities providing EmONC in Africa, Nigeria inclusive [13]. EmONC training is said to be included in 65% of the interventions to reduce maternal and newborn deaths [14,15] and previous studies affirmed EmONC training in Nigeria [8,15–17]. Despite the training, the quality of EmONC has been poor [12,18] and efforts to improve the quality has yielded a modest result [19,20] which implies there may be gaps in the implementation of EmONC which has not been documented. For instance, the extent to which EmONC has become part of routine work in the healthcare facilities is rarely documented in the literature.

Integration of EmONC is necessary for EmONC effectiveness to avert maternal and newborn deaths that result from obstetric complications. Integration is an implementation outcome that focuses on the extent to which an intervention has truly and in reality become part of work in services or organization [21]. Evidence has shown that failure in the integration of evidence-based intervention to routine practice results in the failure of patients receiving quality care [22]. The quality of care relates to the failure to receive adequate and appropriate care when women with obstetric complications arrive healthcare facilities which result in preventable deaths for these women and their newborn.

While EmONC is not a newly adopted intervention in Nigeria, the anticipated result has not been realised. This is evident in the country’s maternal and mortality ratio of 814 /100,000 deaths and neonatal mortality ratio of 34/1,000 births [2]. There is also a growing consensus among scholars on the need to focus on implementation research to promote positive health outcome [23,24]. Implementation research on the integration of EmONC is invaluable to know the extent to which care for obstetric complications to avert preventable deaths is readily provided in healthcare facilities in Nigeria. Previous studies have focussed on effectiveness, availability, quality and use of EmONC [4,6,8,18]. While these efforts are commendable, they have not produced effective strategies to solve the challenges with EmONC as high maternal and neonatal mortality from perinatal complications persist in Nigeria.

Assessing integration on an established framework of normalization process theory offers the opportunity for comparability and ensures generalizability of knowledge from various local context [25]. To a large extent, studies on EmONC in Nigeria have not addressed the implementation outcome such as integration at all levels of healthcare facilities where EmONC is assumed to be implemented. This paper, as part of a larger study on the implementation of EmONC focused on the assessment of integration of EmONC in maternal and newborn care (MNC) in the public healthcare facilities from the report of service providers. In Nigeria, only 36% of births are facility-based and about two-third of this occur in public healthcare facilities [26,27]. The public healthcare facilities are funded by the government and expected to be staffed with qualified healthcare workers who should have received EmONC training. The assessment of integration makes this paper fulfils one important research agenda for advancing implementation science in terms of the conceptualization and measurement of implementation outcome [21].

## Materials and methods

This is a descriptive cross-sectional study conducted at the three levels of healthcare delivery system in Osun State, Nigeria. Osun State is one of the 36 states in Nigeria. The state has three senatorial districts, six administrative zones and thirty Local Government Areas (LGAs). A purposive sampling technique was used to select nine of the thirty LGAs (the three LGAs with the three tertiary healthcare facilities in the state and another six LGAs with State hospitals in the six administrative zones of the state). Also, four primary healthcare facilities in each of the six LGAs where State hospitals are located were selected. Therefore, a total of 33 health facilities (three tertiary, six secondary and twenty-four primary healthcare facilities) were selected based on World Health Organisation framework of linking one CEmONC healthcare facility to four BEmONC healthcare facilities [9]. All 505 healthcare providers and facility managers who were expected to be involved in the implementation of EmONC in the 33 healthcare facilities gave their consent to participate in the study. These include BEmONC service providers at the primary healthcare facilities and both BEmONC and CEmONC service providers at the secondary and tertiary healthcare facilities. From the record, majority of the healthcare providers working in the primary healthcare facilities are community health workers (community health extension workers (CHEWs), community health officers (CHOs) and health assistants) with few nurse-midwives (usually a nurse-midwife or nurse per facility) and one medical practitioner (general practitioner, non-specialist) over-seeing all the primary healthcare facilities in a LGA [28] which could be up to fifteen healthcare facilities. However, the voluntary community health workers that were working in the public healthcare facilities, who were not employed by the government were excluded from the study. In secondary and tertiary healthcare facilities, the healthcare providers are nurse/midwives and medical practitioners with good proportions as specialists in the tertiary healthcare facilities. The instrument used for measuring EmONC integration was an adapted questionnaire from NoMad instrument. NoMad instrument measures implementation process from the perspective of healthcare providers who were involved in the implementation process [29] and was theoretically derived from the Normalization process theory [30]. The questionnaire consisted of two parts; the first part assessed the socio- demographic and work-related characteristics of the respondents in relation to implementation of EmONC. The second part of the questionnaire assessed the healthcare providers’ report of integration of EmONC with 20 question items (coherence (4), cognitive participation (4), collective action (7) and reflexive monitoring (5)) on a 5-point Likert scale of agreement for response (strongly agree, agree, neither agree nor disagree, disagree and strongly disagree). These response categories were as specified by the NoMad instrument. The reliability of the questionnaire was established in a pilot study with a Cronbach alpha of 0.95. The validity of the questionnaire was established through face and content validity criteria. Ethical approval for the study was obtained from the Health Research Ethics Committee (HREC) of the Institute of Public Health, Obafemi Awolowo University, Ile-Ife, Ethics and Research Committee, Obafemi Awolowo University Teaching Hospital Complex, Ile-Ife and informed consents taken from the participants. The data were entered into APHRC REDCap and analysed using STATA 14. For the 20 question-items which measure four mechanisms of integration on 5-point Likert scale, a score of 1 was allotted to ‘strongly disagree’, 2 to ‘disagree’, 3 to ‘neither agree nor disagree’, 4 to ‘agree’ and 5 to ’strongly agree’ for positively worded questions and vice versa for negatively worded questions. Attention to details considering the test items was done to pick critical details that can be deduced from each of the item and the integration score was transformed to continuous variable as 100% and the responses of respondents on integration expressed as percentage (%). Thus, the extent of integration was considered at two levels, at the item by item level and in percentage (%). Univariate analysis was done using frequency, percentage, median and means. Bivariate analysis was done using Kruskal Wallis test to determine difference in EmONC integration in primary, secondary and tertiary healthcare facilities while Mann Whitney test was used to determine the difference in EmONC integration among healthcare providers who had EmONC training and those who had EmONC guidelines available on their wards and units. Mann Whitney test was used because EmONC integration was skewed to the right and the independent variables (EmONC training and EmONC guidelines) were dichotomous.

## Results

### Socio-demographic and occupational characteristics of healthcare providers

Table 1 shows the socio-demographic and occupational characteristics of the healthcare providers and facility managers. The mean age of healthcare providers was 38.68±8.27. A large proportion of the respondents were female, and 66.5% had a diploma certificate. Half of the respondents (52.5%) were community health extension workers (CHEWs). About two-thirds of respondents (63.2%) were from primary healthcare facilities and (62.6%) have spent up to 3 years in the maternity unit. A high percentage (70.7% & 77.6%) of respondents did not have training in EmONC and also reported non-availability of EmONC guidelines respectively.

**Table 1:** Respondents’ socio-demographic and occupational characteristics (n = 505).

Variables	Frequency	Percentage (%)
**Age: Mean = 38.68±8.27**		
20–29	77	15.2
30–39	197	39.0
40–49	182	36.0
50 and above	49	9.7
**Sex**		
Male	51	10.1
Female	454	89.9
**Educational level**		
Diploma	336	66.5
First degree	150	29.7
Master	16	3.2
PhD	1	0.2
Fellowship	2	0.4
**Job category**		
Facility manager	21	4.1
Medical practitioner	62	12.3
Nursing staff	157	31.1
Community health workers	265	52.5
**Level of healthcare facility**		
Primary	319	63.2
Secondary	49	9.7
Tertiary	137	27.1
**Years spent on the in the maternity unit**		
Less than one year	119	23.6
1–3 years	197	39.0
4–6 years	81	16.0
7–9 Years	30	5.9
10 years and above	78	15.5
**Training on EmONC**		
Yes	148	29.3
No	357	70.7
**Availability of EmONC guidelines in the maternity unit**		
Yes	392	22.4
No	113	77.6

### Mechanism of EmONC integration in primary healthcare facilities

Table 2 shows the mechanism of integration in primary healthcare facilities. The table shows in the domain of coherence, a large percentage of respondents 86.8% (35.4%+51.4%) agreed that EmONC differs from usual MNC while 87.5% (29.5%+58.0%) agreed that they can see the potential value of EmONC. Also, in the domain of cognitive participation, majority of the respondents 93.4% (40.1%+53.3%) agreed that they will continue to support EmONC while 78.1% (27.9%+50.2%) agreed EmONC is a legitimate part of their role. In the domain of collective action, 59.2% (23.8%+35.4%) agreed that sufficient training was provided for implementation of EmONC, 45.1% (16.6%+28.5%) agreed that sufficient resources were available to support EmONC while 59.6% (20.4%+39.2%) agreed that management adequately supports EmONC. The table also shows in the domain of reflexive monitoring, 88.7% (26.0%+62.7%) agreed that EmONC is worthwhile and 89.4% (32.0%+57.4%) agreed that feedback on EmONC can be used to improve EmONC.

**Table 2:** Mechanism of integration of EmONC into maternal and newborn care in primary healthcare facilities (n = 319).

Variables	SA Freq (%)	A Freq (%)	NAND Freq (%)	DS Freq (%)	SD Freq (%)
**Coherence**					
EmONC differs from usual maternal and neonatal care	113 (35.4)	164 (51.4)	18 (5.6)	16 (5.0)	8 (2.5)
Staff have an understanding of the purpose of EmONC	64 (20.1)	210 (65.8)	15 (4.7)	24 (7.5)	6 (1.9)
I understand how EmONC affects my work	60 (18.8)	144 (45.1)	41 (12.9)	59 (18.5)	15 (4.7)
I can see the potential value of EmONC for my work	94 (29.5)	185 (58.0)	21(6.6)	15 (4.7)	4 (1.3)
**Cognitive participation**					
Key people drive EmONC forward and get others involved	71 (22.3)	167 (52.4)	25 (7.8)	47 (14.7)	9 (2.8)
Participating in EmONC is a legitimate part of my role	89 (27.9)	160 (50.2)	18 (5.6)	44 (13.8)	8 (2.5)
I’m open to working with colleagues in new ways to deliver EmONC	93 (29.2)	175 (54.9)	20 (6.3)	27 (8.5)	4 (1.3)
I will continue to support EmONC	128 (40.1)	170 (53.3)	8 (2.5)	10 (3.1)	3 (0.9)
**Collective action**					
I can easily integrate EmONC into my existing work	82 (25.7)	158 (49.5)	22(6.9)	52 (16.3)	5 (1.6)
EmONC disrupts working relationships	28 (8.8)	79 (24.8)	28 (8.8)	149 (46.7)	35 (11.0)
Confident in other people’s ability to deliver EmONC	58 (18.2)	186 (58.3)	34 10.7)	33 (10.3)	8 (2.5)
Work assigned to those with appropriate EmONC skills	104 (32.6)	171 (53.6)	13(4.1)	23 (7.2)	8 (2.5)
Sufficient training is provided to implement EmONC	76 (23.8)	113 (35.4)	16 (5.0)	85 (26.6)	29 (9.1)
Sufficient resources are available to support EmONC	53 (16.6)	91 (28.5)	25(7.8)	98 (30.7)	52 (16.3)
Management adequately supports EmONC	65 (20.4)	125 (39.2)	21 (6.6)	68 (21.3)	40 (12.5)
**Reflexive monitoring**					
I am aware of reports about the effects of EmONC	72 (22.6)	171 (53.6)	18 (5.6)	43 (13.5)	15 (4.7)
The staff agree that EmONC is worthwhile	83 (26.0)	200 (62.7)	18 (5.6)	14 (4.4)	4 (1.3)
I value the effects that EmONC has on my work	74 (23.2)	198 (62.1)	23 (7.2)	17 (5.3)	7 (2.2)
Feedback about EmONC can be used to improve it	102 (32.0)	183 (57.4)	14 (4.4)	15 (4.7)	5 (1.6)
I can modify how I work with EmONC	62 (19.4)	178 (55.8)	30 (9.4)	40 (12.5)	9 (2.8)

**Key—SA** strongly agree; **A** agree; **NAND** neither agree nor disagree; **D** disagree; **SD** strongly disagree.

### Mechanism of EmONC integration in secondary healthcare facilities

Table 3 shows the mechanism of integration in secondary healthcare facilities. The table shows in the domain of coherence, a large percentage of respondents 77.5% (46.9%+30.6%) agreed that EmONC differs from usual MNC while 81.6% (59.2%+14.3%) agreed that they can see the potential value of EmONC. Also, in the domain of cognitive participation, majority of the respondents 89.8% (59.2%+30.6%) agreed that EmONC is a legitimate part of their role while 87.8% (49.0%+38.8%) agreed that they will continue to support EmONC. In the domain of collective action, 55.1% (28.6%+26.5%) agreed that sufficient training was provided for implementation of EmONC, 34.7% (18.4%+16.3%) agreed that sufficient resources were available to support EmONC while 36.8% (28.6%+8.2%) agreed that management adequately supports EmONC. The table also shows in the domain of reflexive monitoring, 63.3% (44.9%+18.4%) agreed they were aware of EmONC effects, 75.5% (63.3%+12.2%) agreed that EmONC is worthwhile, and 89.8% (46.9%+42.9%) agreed that feedback on EmONC can be used to improve EmONC.

**Table 3:** Mechanism of integration of EmONC into maternal and newborn care in secondary healthcare facilities (n = 49).

Variables	SA Freq (%)	A Freq (%)	NAND Freq (%)	DS Freq (%)	SD Freq (%)
**Coherence**					
EmONC differs from usual maternal and neonatal care	23 (46.9)	15 (30.6)	4 (8.2)	5 (10.2)	2 (4.1)
Staff have an understanding of the purpose of EmONC	29 (59.2)	7 (14.3)	3 (6.1)	9 (18.4)	1(2.0)
I understand how EmONC affects my work	24 (49)	4 (8.2)	9(18.4)	7 (14.3)	5 (10.2)
I can see the potential value of EmONC for my work	29 (59.2)	11 (22.4)	6 (12.2)	2 (4.1)	1(2.0)
**Cognitive participation**					
Key people drive EmONC forward and get others involved	32(65.3)	10 (20.4)	4 (8.2)	2 (4.1)	1 (2.0)
Participating in EmONC is a legitimate part of my role	29 (59.2)	15 (30.6)	4 (8.2)	0 (0)	1(2.0)
I’m open to working with colleagues in new ways to deliver EmONC	24 (49.0)	19 (38.8)	4 (8.2)	1 (2.0)	1 (2.0)
I will continue to support EmONC	24 (49.0)	19 (38.8)	4 (8.2)	1 (2.0)	1 (2.0))
**Collective action**					
I can easily integrate EmONC into my existing work	23 (46.9)	13 (26.5)	5 (10.2)	8 (16.3)	0 (0)
EmONC disrupts working relationships	5 (10.2)	8 (16.3)	9 (18.4)	17 (34.7)	10 (20.4)
Confident in other people’s ability to deliver EmONC	29 (59.2)	6 (12.2)	7 (14.3)	6 (12.2)	1 (2.0)
Work assigned to those with appropriate EmONC skills	18 (36.7)	15 (30.6)	11 (22.4)	5 (10.2)	0 (0)
Sufficient training is provided to implement EmONC	14 (28.6)	13 (26.5)	7(14.3)	10 (20.4)	5(10.2)
Sufficient resources are available to support EmONC	9 (18.4)	8 (16.3)	16 (32.7)	10 (20.4)	6 (12.2)
Management adequately supports EmONC	14 (28.6)	4 (8.2)	16 (32.7)	11 (22.4)	4(8.2)
**Reflexive monitoring**					
I am aware of reports about the effects of EmONC	22 (44.9)	9 (18.4)	12 (24.5)	4(8.2)	2(4.1)
The staff agree that EmONC is worthwhile	31 (63.3)	6 (12.2)	6 (12.2)	5 (10.2)	1 (2.0)
I value the effects that EmONC has on my work	24 (49)	7 (14.3)	13 (26.5)	3 (6.1)	2(4.1)
Feedback about EmONC can be used to improve it	23 (46.9)	21 (42.9)	3 (6.1)	1 (2.0)	1 (2.0)
I can modify how I work with EmONC	23 (46.9)	12 (24.5)	10 (20.4)	3(6.1)	1 (2.0)

**Key—SA** strongly agree; **A** agree; **NAND** neither agree nor disagree; **D** disagree; **SD** strongly disagree.

### Mechanism of EmONC integration in tertiary healthcare facilities

Table 4 shows the mechanism of integration in tertiary healthcare facilities. The table shows in the domain of coherence, a large percentage of respondents 80.3% (53.3%+27.0%) agreed EmONC differs from usual MNC while 92.7% (67.2%+25.5%) agreed they can see the potential value of EmONC. Also, in the domain of cognitive participation, majority of the respondents 89.8% (51.1%+38.7%) agreed EmONC is a legitimate part of their role while 93.4% agreed they will continue to support EmONC. In the domain of collective action, 55.5% (30.7%+24.8%) agreed that sufficient training was provided for implementation of EmONC, 41.6% (29.2%+12.4%) agreed that sufficient resources were available to support EmONC while 61.3% (50.4%+10.9%) agreed that management adequately supports EmONC. The table also shows in the domain of reflexive monitoring, 71.5% (48.9%+22.6%) agreed they were aware of EmONC effects, 81.8% (57.7%+24.1%) agreed that EmONC is worthwhile, and 93.4% (54.0%+39.4%) agreed that feedback on EmONC can be used to improve EmONC.

**Table 4:** Mechanism of integration of EmONC into maternal and newborn care in tertiary healthcare facilities (n = 137).

Variables	SA Freq (%)	A Freq (%)	NAND Freq (%)	DS Freq (%)	SD Freq (%)
**Coherence**					
EmONC differs from usual maternal and neonatal care	73 (53.3)	37 (27.0)	13(9.5)	10 (7.3)	4 (2.9)
Staff have an understanding of the purpose of EmONC	78 (56.9)	32 (23.4)	13 (9.5)	7(5.1)	7 (5.1)
I understand how EmONC affects my work	82 (59.9)	33 (24.1)	13 (9.5)	5 (3.6)	4 (2.9)
I can see the potential value of EmONC for my work	92 (67.2)	35 (25.5)	7 (5.1)	2 (1.5)	1(0.7)
**Cognitive participation**					
Key people drive EmONC forward and get others involved	79 (57.7)	42 (30.7)	9 (6.6)	6 (4.4)	1(0.7)
Participating in EmONC is a legitimate part of my role	70 (51.1)	53 (38.7)	10 (7.3)	2 (1.5)	2 (1.5)
I’m open to working with colleagues in new ways to deliver EmONC	64 (46.7)	60 (43.8)	8 (5.8)	2(1.5)	3 (2.2)
I will continue to support EmONC	60 (43.8)	68 (49.6)	5 (3.6)	3 (2.2)	1 (0.7)
**Collective action**					
I can easily integrate EmONC into my existing work	78 (56.9)	42 (30.7)	13 (9.5)	3 (2.2)	1 (0.7)
EmONC disrupts working relationships	7 (5.11)	23 (16.8)	8 (5.8)	70 (51.1)	29 (21.2)
Confident in other people’s ability to deliver EmONC	75 (54.7)	14 (10.2)	28 (20.4)	18 (13.1)	2 (1.5)
Work assigned to those with appropriate EmONC skills	79 (57.7)	27 (19.7)	12 (8.8)	14 (10.2)	5(3.6)
Sufficient training is provided to implement EmONC	42 (30.7)	34 (24.8)	28 (20.4)	27 (19.7)	6 (4.4)
Sufficient resources are available to support EmONC	40 (29.2)	17 (12.4)	29 (21.2)	33 (24.1)	18 (13.1)
Management adequately supports EmONC	69 (50.4)	15 (10.9)	22 (16.1)	23 (16.8)	8 (5.8)
**Reflexive monitoring**					
I am aware of reports about the effects of EmONC	67 (48.9)	31 (22.6)	22 (16.1)	14 (10.2)	3 (2.2)
The staff agree that EmONC is worthwhile	79 (57.7)	33 (24.1)	15 (10.9)	6 (4.4)	4 (2.9)
I value the effects that EmONC has on my work	76 (55.5)	37 (27)	17 (12.4)	6 (4.4)	1 (0.7)
Feedback about EmONC can be used to improve it	74 (54.0)	54 (39.4)	4(2.9)	4 (2.9)	1 (0.7)
I can modify how I work with EmONC	87 (63.5)	24 (17.5)	19 (13.9)	4(2.9)	3 (2.2)

**Key—SA** strongly agree; **A** agree; **NAND** neither agree nor disagree; **D** disagree; **SD** strongly disagree.

### Integration of EmONC in primary, secondary and tertiary healthcare facilities

**Fig 1:**
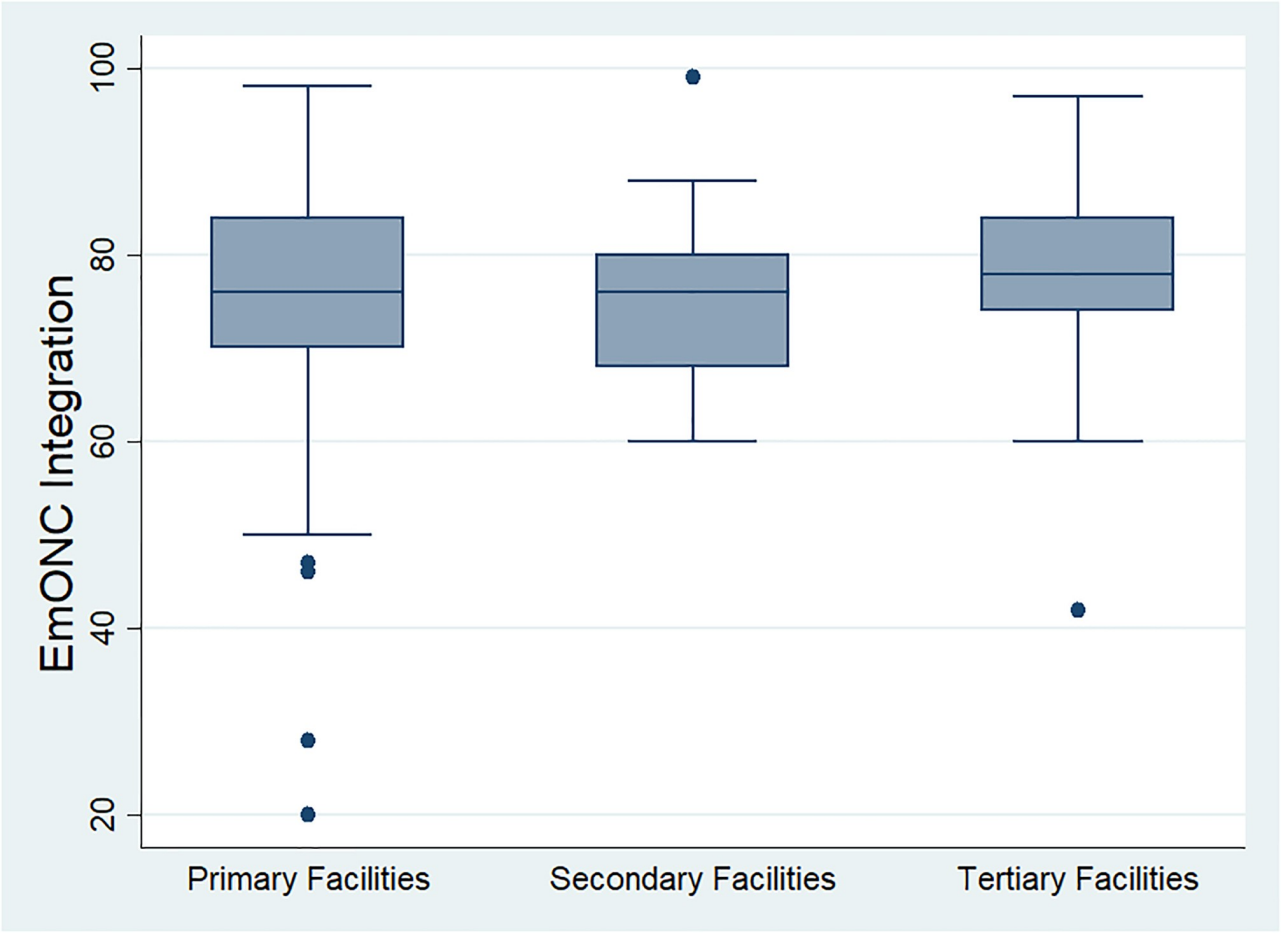
Integration of EmONC by the level of healthcare facility.

In Fig 1, the box and whisker plot shows the integration of EmONC at the three levels of healthcare delivery. The percentage integration score at the primary healthcare facilities was 76 (IQR = 84–70), 76 (IQR = 80–68) at secondary healthcare facilities, and 78 (IQR = 84–74) at the tertiary healthcare facilities. The outliers in the primary healthcare facilities indicate some extremely low scores of integration, as low as a score of 20%.

Integration was highest at the tertiary healthcare facilities with a median score of 78 and narrowest total variation. The extent of EmONC integration at primary and secondary healthcare facilities was the same (76) although primary healthcare facilities had a wider total variation.

### The difference in primary, secondary and tertiary healthcare facilities by the mechanism of integration

Table 5 shows the Kruskal Wallis test to determine the difference between primary, secondary and tertiary healthcare facilities on the four domains of the mechanism of integration. There was a statistical difference in the domain of coherence at the three levels of health care (H (2) = 8.562, p = 0.013), with a mean rank of 254 for the primary healthcare facilities, 199 for the secondary healthcare facilities and 270 for tertiary healthcare facilities. Also, there was a statistical difference in the domain of cognitive participation at the three levels of healthcare (H (2) = 13.935, p = 0.001), with a mean rank of 236 for the primary health facilities, 260 for the secondary healthcare facilities and 291 for tertiary healthcare facilities. The result shows there was a higher (better) coherence and cognitive participation in EmONC among the healthcare providers in tertiary healthcare facilities than those in primary and secondary healthcare facilities. However, the healthcare providers in primary healthcare facilities had better coherence than those in secondary healthcare facilities while those in secondary healthcare facilities were better in cognitive participation for EmONC.

**Table 5:** Difference between levels of healthcare facilities by the mechanism of EmONC integration (n = 505).

Domains	Rank Sum Primary 319	Secondary 49	Tertiary 137	x^2^	P
Coherence	81030.00	9753.00	36982.00	8.562	0.013*
Cognitive participation	75171.50	12716.50	39877.00	13.935	0.001*
Collective action	80306.50	11168.00	36290.50	2.381	0.304
Reflexive monitoring	81114.00	10846.50	35804.50	2.777	0.243

### Integration of EmONC by EmONC training and guidelines

**Fig 2:**
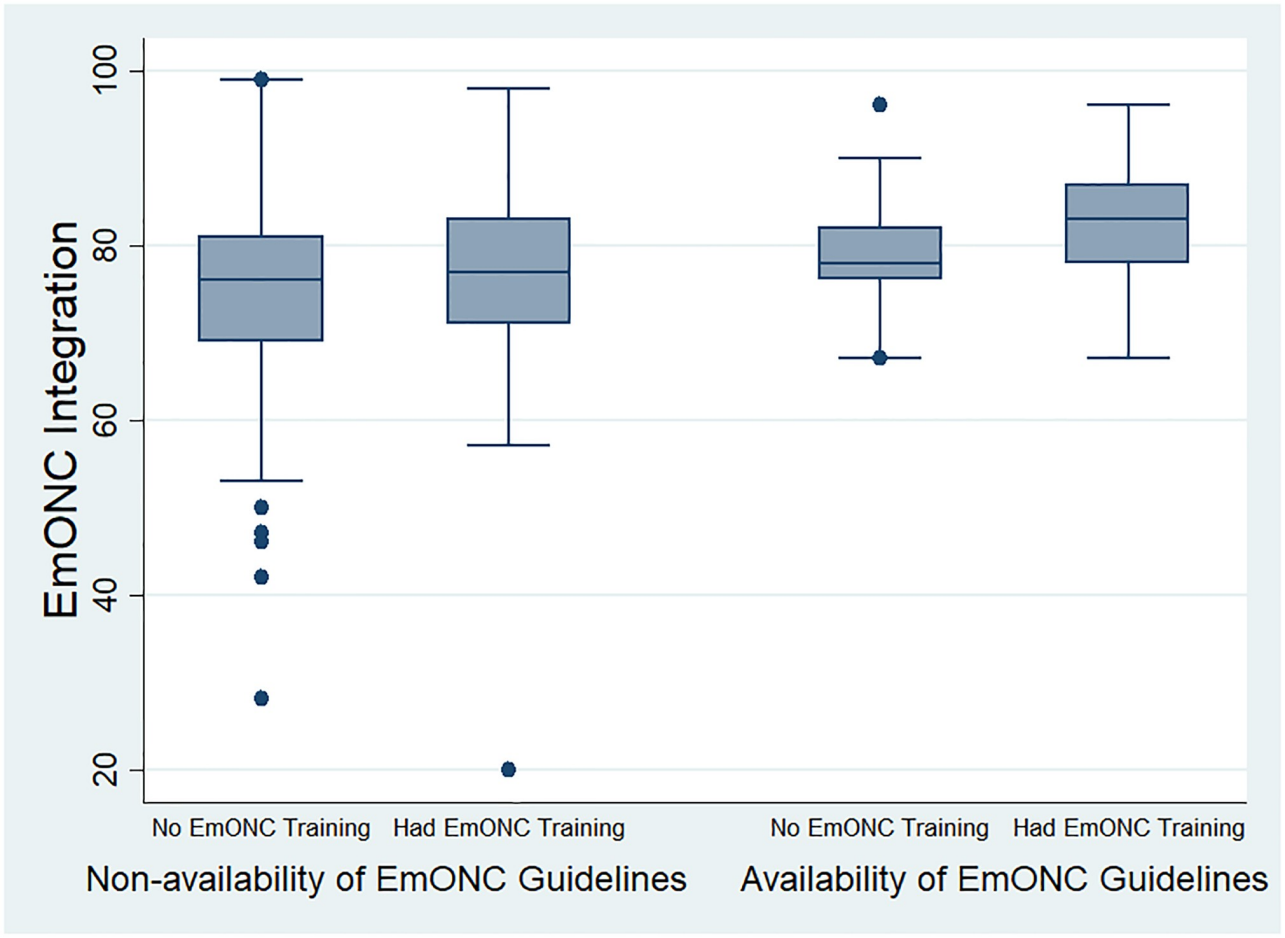
Integration of EmONC by training and guidelines.

In Fig 2, the box and whisker plot shows the integration of EmONC with training and guidelines availability. The integration score for healthcare providers who had no EmONC training and no EmONC guidelines available to them was 76 (IQR = 81–69) while the integration score for healthcare providers who had EmONC training but had no EmONC guidelines was 77 (IQR = 83–71). The integration score with the category of healthcare providers who had no EmONC training but had EmONC guidelines was 78 (IQR = 82–76) while the integration score for healthcare providers who had EmONC training and also had EmONC guidelines available was 83 (IQR = 87–78). The figure shows EmONC integration was highest among healthcare providers with EmONC training and guidelines.

### EmONC training and guideline availability in healthcare facilities

Table 6 shows the percentage distribution of healthcare providers with EmONC training and guidelines in primary, secondary and tertiary healthcare facilities. Majority of the respondents (75.9% in primary facilities, 81.6% in secondary facilities) did not have EmONC training. Also, 86.5% of respondents in primary facilities and 81.6% in secondary facilities did not have EmONC guideline available in the maternity unit where they work. The table also shows 54.7% and 55.5% of respondents in tertiary facilities did not have EmONC training and also did not have guidelines available respectively.

**Table 6:** Percentage distribution of respondents with EmONC training and guidelines in healthcare facilities.

Variable	Primary facilities Freq (%)	Secondary facilities Freq (%)	Tertiary facilities Freq (%)
EmONC Training			
Had EmONC Training	77 (24.1)	9 (18.4)	62 (45.3)
No EmONC Training	242 (75.9)	40 (81.6)	75 (54.7)
Availability of EmONC Guideline			
EmONC Guideline available	43 (13.5)	9 (18.4)	61 (44.5)
EmONC Guideline not available	276 (86.5)	40 (81.6)	75 (55.5)

### Difference in integration by levels of healthcare facilities, EmONC training and guidelines

Table 7 displays the Mann Whitney test which indicated that there was a statistically significant difference in EmONC integration between healthcare providers who had EmONC training and those that did not have EmONC training (p = 0.001). The table also shows a statistically significant difference in EmONC integration between healthcare providers who had EmONC guidelines and those that did not have EmONC guidelines (p = 0.001).

**Table 7:** Difference in EmONC integration by EmONC training and guideline availability.

Independent Variable	N	Rank sum	p-value
**EmONC Training**			
Had EmONC Training	148	44439	0.001
No EmONC Training	357	83326	
**Availability of EmONC Guideline**			
EmONC Guideline available	113	36834.5	0.001
EmONC Guideline not available	392	90930.5	

Table 8 presents the Kruskal Wallis text which shows that there was a statistically significant difference in the integration of EmONC among healthcare providers at the three levels of healthcare delivery (H (2) = 6.146, p = 0.046). with a mean rank of 248 for the primary healthcare facilities, 220 for the secondary healthcare facilities and 276 for tertiary healthcare facilities. The result shows there was a higher EmONC integration among the healthcare providers in tertiary healthcare facilities than those in primary and secondary healthcare facilities.

**Table 8:** Difference in EmONC integration by level of healthcare facility (n = 505).

Level of Healthcare Facility	N	Rank sum	x^2^	p-value
Primary	319	79199.50	6.146	0.046
Secondary	49	10789.50		
Tertiary	137	37776.00		

## Discussion

Implementation research assessing the integration of EmONC is important in bridging the gap between the evidence of EmONC effectiveness and its real-world practice. The integration of evidence-based practice is dependent on the collective and coordinated behaviour of health professionals within the complex healthcare system [29]. Integration of EmONC among healthcare providers and facility managers is through a mechanism that ensures collectiveness and coordination. This study assessed the mechanism and extent of integration of EmONC among the health care providers and facility managers based on their report using normalization process theory.

The study was carried out among five hundred and five (505) healthcare providers and facility managers in primary, secondary and tertiary healthcare facilities in Osun State. The categories of healthcare providers in this study were the nursing staff (nurses/midwives), medical practitioners, and community health extension workers (CHEWs). More than half of the respondents were working in primary healthcare facilities. The World Health Organisation recommendation to link four basic EmONC healthcare facilities (primary healthcare facilities) to one comprehensive EmONC healthcare facilities [9] necessitated that more primary healthcare facilities be involved in the study. However, previous studies allude to the fact that the primary healthcare facilities in Nigeria are majorly staffed with community health workers (CHOs, CHEWs and health assistants) who are not professionally trained and qualified to provide care for women with obstetric complications [28,31]. In a study carried out in primary healthcare facilities in the southwest, Nigeria, 68.5% of staff providing care are community health workers [28]. Many of the healthcare providers in the primary healthcare facilities are community health workers and this explains the preponderance of community health workers in the study. The implication of staffing primary healthcare facilities majorly with community health workers who are not professionally qualified and incompetent to provide EmONC is grave. There are numerous unnecessary referrals to higher levels of care (secondary and tertiary healthcare facilities) with increasing likelihood of mortality [32] as many women who should have received care from competent medical practitioners and nurse-midwives at the primary healthcare facilities are referred out. This culminate in non-patronage of primary healthcare facilities for maternity care and self-referral to higher levels of care [33,34]. There are therefore, many ‘normal’ cases of obstetrics that do not require specialist care in secondary and tertiary healthcare facilities which further stresses these facilities.

A review of the minimum standards for primary healthcare shows basic emergency obstetric care should be provided for women with obstetric complications by a medical officer (where available) and nurse/midwife, while newborn resuscitation should be provided by a medical officer, nurses/midwife, CHO and CHEW [35]. The staff structure in primary healthcare facilities in which majority of the healthcare providers are community health workers [28,31] implies there is no provision by Nigeria standard to make EmONC fully integrated into MNC in primary healthcare facilities. This is because nurses and doctors that would implement EmONC are very few in primary healthcare facilities. The unpredictability of obstetric complications still put many community health workers in a position to implement EmOC, despite their lack of competence to do this and with the observation that EmOC is not a legitimate part of their work. These observations notwithstanding, this study still showed a report of high integration of EmONC in primary healthcare facilities, reflecting inconsistencies in report by healthcare providers and realities at the primary healthcare facilities.

A careful comparison of the mechanisms of integration across the three levels of healthcare delivery shows there was a difference in the mechanism of integration between primary, secondary and tertiary healthcare facilities. The findings of the study reveal that there was a difference in coherence and cognitive participation across the three levels of healthcare delivery. This finding implies that the stakeholders’ (healthcare providers and facility managers) understanding of the tasks that the implementation of EmONC requires of them and involvement in EmONC differs across the primary, secondary and tertiary healthcare facilities. This finding is expected as healthcare providers in primary healthcare facilities provide BEmONC while those in secondary and tertiary healthcare facilities provide both BEmONC and CEmONC [9]. On the other hand, the non-patronage of primary healthcare facilities and self-referral to higher levels of care affirmed there was a difference in the involvement of the healthcare providers across the three levels of healthcare [33,34]. Many healthcare providers and facility managers especially the community health workers which are the majority in the primary healthcare facilities refer out cases of obstetric emergencies that require EmONC because the management for these cases was not included in the standing order which is a guide for their practice. It is significant that EmONC could not have been included in the standing order for this category of service providers as this relate to their level of training and competencies.

The findings established that EmONC has not been fully integrated into maternal and newborn care at all levels of healthcare delivery in the state. The results show there was a difference in the level of integration across primary, secondary and tertiary healthcare facilities. Integration of EmONC is higher in the tertiary healthcare facilities than in primary and secondary healthcare facilities. The tertiary healthcare facilities are referral centres with multidisciplinary experts in obstetrics care and management of complications. However, there is minimal collective action for EmONC integration at all levels of health care delivery as the findings of the study suggest sufficient training is not provided to staff for the implementation of EmONC, sufficient resources are not available and management does not adequately support EmONC implementation at all levels of healthcare delivery. Previous studies in Nigeria affirmed that the quality of EmONC is poor [12,18] and efforts to improve the quality of EmONC [19,20] yielded a modest result. The minimal collective action for EmONC at the three levels of healthcare facilities as revealed by the findings of this study could be responsible for the poor quality of EmONC claimed by previous studies and the consequent sub-optimal pregnancy outcome in Nigeria.

The integration of EmONC at the healthcare facilities was associated with EmONC training and availability of EmONC guidelines in the maternity units. Majority of respondents in the primary and secondary healthcare facilities did not have training in EmONC nor have guidelines available in the maternity unit where they work. Healthcare providers who have been trained on the implementation of EmONC and also have access to EmONC guidelines are more likely to higher integration than those without training and guidelines. Lack of training and unavailability of guidelines may impact negatively on the knowledge, skills and confidence of healthcare providers to implement EmONC. Previous studies conducted in Nigeria documented poor knowledge and skills of health care providers [20,36] which may relate to lack of sufficient training which negatively impacts confidence to implement EmONC and the failure to fully integrate EmONC at primary, secondary and tertiary healthcare facilities. The high level of EmONC integration seen in this study may have been because many healthcare providers in primary healthcare facilities who are not qualified to implement EmOC by the minimum standard of primary healthcare indicated the implementation of EmONC is their legitimate role. It may also be related to the unpredictability of the occurrence of obstetric complications which makes this category of healthcare provider to engage in the management of these complications.

Though EmONC integration seems to be high in all the healthcare facilities, it may be related to the fact that the study was done in public healthcare facilities where full MNC such as antenatal, intranatal and postnatal care are implemented. This was necessary to ensure that respondents have practical experience of care for obstetric complications as this study was part of a larger study on implementation of EmONC. So, exclusion of public healthcare facilities that only offer partial MNC might have contributed to the high EmONC integration among healthcare providers in this study. Also, this was a reported integration, and may not have reflected the actual integration of EmONC in the healthcare facilities. A complementary qualitative approach would be necessary to validate the high integration reported in this study.

The state of maternal and newborn health as well as the high maternal and newborn mortality in Nigeria posed a query on the high EmONC integration and the authenticity of the information given by the respondents. Evidence has shown that the inability to implement effective interventions in healthcare facilities occurs because such interventions are not integrated into existing programmes [22]. The implementation of EmONC at primary, secondary and tertiary healthcare facilities is expected to yield a positive outcome of healthy mothers and babies and reduction in maternal and newborn mortality.

Considering the items in the mechanism of integration, many of the healthcare providers concluded the management of the hospitals were not supportive enough with regards to EmONC implementation. The lack of training and guidelines as widely reported in this study as well as insufficient resources to implement EmONC is a clog in the wheels of successful implementation of EmONC in healthcare facilities. Diffin et al. noted that interventions are likely to be successfully implemented if there is an opportunity to implement them [37]. Lack of support for EmONC implementation in healthcare facilities may mean some women with obstetric complications and their babies will not receive adequate and appropriate care, which further increases the maternal and newborn morbidity and mortality in Nigeria. There is also the possibility of non-patronage of public healthcare facilities by many women who would have benefited from EmONC if they had delivered in healthcare facilities. The perception of “too much referral” to higher levels of care for what could have been done at the primary care level may send a wrong signal about the situation and lead to non-patronage of facility-based care. Previous study affirmed that women’s perception of facility-based care as ‘medicalized’ hinders them from patronizing healthcare facilities [38]. Therefore, government and non-governmental organisations need to provide adequate support and resources needed to get EmONC fully integrated into routine MNC at all levels of care.

It needs to be mentioned that the assumption that services at the primary care levels do not have to be provided by practitioners with desirable competencies that can assure better access to quality EmONC and MNC as operational in many public healthcare settings in Nigeria has negative consequences for healthcare access to clients and undesirable congestions at the secondary and tertiary levels of care. One critical observation by the investigators in this study is the lack of monitoring, evaluation and communication strategies in the implementation of EmONC. This would also need to be given due attention and further investigations.

## Conclusion

Integration of emergency obstetric and newborn care in maternal and child care has been sparsely studied. This study is one of the pioneering studies assessing the implementation process of EmONC in the context of its integration on an established framework of normalization process theory. Although the quantitative approach may not give the full picture of the reality of integration of EmONC in healthcare facilities, it provided valuable information on the dynamics of implementing complex interventions as it relates to mechanisms for cohesiveness and coordination of individuals involved in getting the interventions integrated in practice. The Normad instrument used has provided some pieces of information on the causes of poor quality of EmONC and where priority for improvement lies. This study had thus provided baseline information on the integration of EmONC using theory oriented quantitative approach with which future studies on EmONC could compare.

The adoption of a theoretical approach for the integration of EmONC as shown in this study, may fail to capture the actual integration required in reality for the effectiveness of EmONC to reduce maternal and newborn mortality. Research on implementation of interventions and programmes in maternal and child health would benefit more from mixed methods research.

### Limitation of the study

There is a dearth of studies for comparison with the findings of this study. Efforts have been made by researchers to assess the integration of evidence-based interventions in well-established programs, though not in Nigeria [22,25], but the context differs in that none of them is on EmONC, thus posing a limitation for comparison. Also, the study is limited in terms of implementation of EmONC in private hospitals. Future research should focus on the implementation of EmONC in private hospitals to provide a holistic view of facility-based care for women and newborns with obstetric complications.
